# Additive–Subtractive Manufacturing Based on Water-Soluble Sacrificial Layer: High-Adhesion Metal Patterning via Inkjet Printing

**DOI:** 10.3390/mi16060706

**Published:** 2025-06-13

**Authors:** Mengyang Su, Jin Huang, Hongxiao Gong, Zihan Zhu, Pan Li, Huagui Wang, Pengbing Zhao, Jianjun Wang, Jie Zhang

**Affiliations:** State Key Laboratory of Electromechanical Integrated Manufacturing of High-Performance Electronic Equipments, Xidian University, Xi’an 710071, China; 23041212582@stu.xidian.edu.cn (M.S.); 23041212620@stu.xidian.edu.cn (Z.Z.); 23041212614@stu.xidian.edu.cn (P.L.); 23041212854@stu.xidian.edu.cn (H.W.); pbzhao@xidian.edu.cn (P.Z.); wangjianjun@xidian.edu.cn (J.W.); jiezhang1987@xidian.edu.cn (J.Z.)

**Keywords:** inkjet printing, adhesion strength, additive–subtractive manufacturing, water-soluble sacrificial layer

## Abstract

Inkjet printing has become a primary technique for manufacturing flexible and conformal electronics due to its digital control, design flexibility, and material compatibility. However, its direct deposition nature results in weak adhesion between metal films and substrates, as it mainly relies on van der Waals or capillary forces, which severely limits its broader application in these fields. To address this limitation, we proposed an additive–subtractive manufacturing method based on a water-soluble sacrificial layer. First, the sacrificial material is inkjet-printed onto the substrate. Then, ion sputtering is employed to bombard the surface with high-energy ions, enabling metal atoms to embed into the substrate and form a strongly adhered conductive layer. Finally, the substrate is immersed in water, dissolving the sacrificial layer and detaching the undesired metal, thereby achieving selective retention of the conductive pattern. Experimental results demonstrate that the optimized water-soluble material, with tailored viscosity and surface tension, enables a patterning resolution of ±10 μm. The adhesion strength of the sputtered metal layer is 5.2 times greater than that of inkjet-printed silver nanoparticles. This method was further applied to fabricate conductive patterns on a curved surface with a 91 mm radius confirming its feasibility and adaptability for complex 3D surfaces.

## 1. Introduction

Inkjet printing, characterized by its low cost, high resolution, and process simplicity, has emerged as a promising additive manufacturing (AM) technique [1,2,3]. Recent advances have broadened its applications across various fields, including flexible sensors used in areas such as disease diagnostics and environmental monitoring [4,5,6,7], energy devices such as solar cells and supercapacitors [8,9,10,11,12], and the large-area fabrication of flexible and stretchable transistors [13,14,15]. These advancements demonstrate inkjet printing’s potential.

However, due to the direct deposition nature of inkjet printing, the adhesion between the metal film and the substrate primarily relies on weak van der Waals forces or capillary forces—the latter existing only before solvent evaporation [16,17,18]. This weak interfacial bonding leads to poor adhesion of the printed conductive patterns, which in turn makes them highly susceptible to cracking, porosity, warping, and even delamination under complex environmental conditions such as high temperature, high humidity, or mechanical stress, severely limiting the broader application of inkjet printing in relevant fields [19,20,21]. Therefore, enhancing the adhesion of inkjet-printed conductive patterns has become a primary focus of research. Xu et al. [22] improved the surface adhesion of dielectric substrates by nearly 6× through surface modification using concentrated sulfuric acid and ultraviolet (UV) laser treatment; however, this approach involves highly corrosive chemicals, raising environmental concerns. Baek et al. [23] achieved a surface modification of polyetheretherketone (PEEK) substrates using concentrated sulfuric acid and potassium permanganate, resulting in a peel-off ratio of less than 0.01% in the adhesion tape test. However, environmentally hazardous chemicals were used and generated during this process. Wang et al. [24] employed a 355 nm laser to modify the surface of polyetheretherketone (PEEK) substrates, achieving a maximum bonding strength of 1.9 MPa—an increase of 413.51% compared to untreated PEEK. Nevertheless, this method is material-specific and limited to PEEK. Li et al. [25] improved surface adhesion via plasma treatment, enabling the fabricated conductive tracks to maintain good electrical conductivity under bending, twisting, and stretching. However, plasma-treated polymer surfaces often undergo “hydrophobic recovery”, wherein the energy of surface-active groups gradually diminishes, typically causing the surface modification effect to degrade within a few hours [26,27]. Jeong et al. [28] developed an inkjet printing method using a well-dispersed aqueous silver ink and a molecular adhesive layer, yielding highly conductive and narrow patterns with strong adhesion to glass substrates; yet, this technique is confined to silver deposition on glass. Similarly, Lee et al. [29] incorporated a silane coupling agent into copper ink to promote adhesion, but this strategy is limited to copper-based inks. Song et al. [30] investigated the influence of printing parameters such as speed and layer number on the microstructure and adhesion of inkjet-printed indium tin oxide (ITO) and In_2_O_3_ films, which are also restricted to specific materials. In summary, current approaches for improving inkjet-printed adhesion suffer from three major limitations: (1) The effect of surface modification is typically short-lived. (2) Many techniques involve corrosive chemicals or high-energy processes, posing environmental and energy-consumption concerns, and (3) most methods suffer from limited versatility, as they are only applicable to specific substrates or conductive materials [31].

This study proposes a hybrid additive–subtractive manufacturing method based on an eco-friendly water-soluble sacrificial material, aiming to enable on-demand fabrication of high-adhesion metal layers on various substrates. First, guided by the Z-number criterion and fluid dynamics modeling, and assisted by a droplet visualization system, a water-soluble sacrificial ink with excellent jetting performance was developed, featuring a viscosity of 8.9 mPa·s and a surface tension of 48 mN/m. Second, surface profilometry revealed a patterning accuracy within ±20 µm. Third, pull-off adhesion tests demonstrated that the optimized process significantly enhanced the interfacial adhesion between the conductive metal layer and the insulating substrate by a factor of 5.2. Finally, well-defined and continuous conductive patterns were successfully fabricated on both a rotating ceramic substrate with a curvature radius of 91 mm and flexible polyethylene glycol terephthalate (PET) films, further demonstrating the feasibility and broad applicability of the proposed method for complex three-dimensional-curved and flexible surfaces.

## 2. Materials and Methods

### 2.1. Additive–Subtractive Hybrid Manufacturing Process

**Figure 1:**
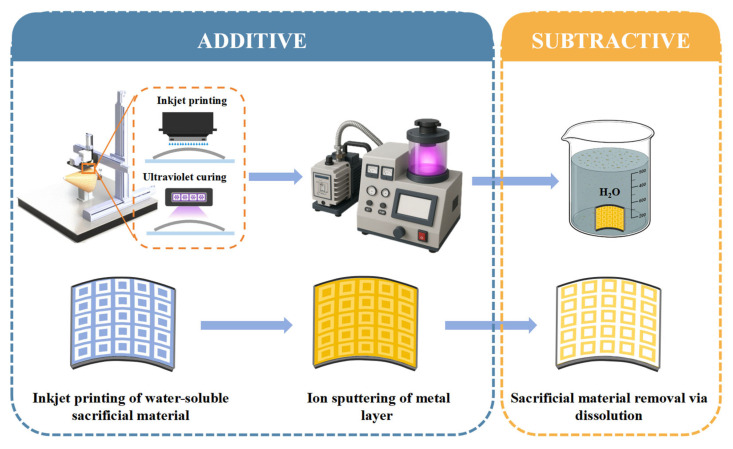
The additive–subtractive hybrid manufacturing process.

The additive–subtractive hybrid manufacturing process proposed in this study is illustrated in Figure 1. First, the substrate is cleaned with an ethanol solution to remove organic contaminants and then pre-dried in an oven at 50 °C for 5 min to ensure a dry and clean surface.

**Figure 2:**
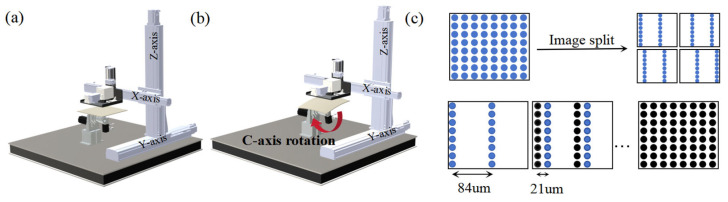
(**a**) Planar printing mode; (**b**) curved printing mode; (**c**) precision printing strategy schematic (the blue dots represent points deposited in the current sub-patterns, the black dots represent points deposited in previous sub-patterns.).

Subsequently, sacrificial patterning is performed using a custom-developed five-axis inkjet printing platform. As shown in Figure 2a,b, the system supports both planar and curved surface printing modes. For flat substrates, the C-axis remains stationary while the printhead moves along the X, Y, and Z axes across the surface. For curved substrates, the C-axis dynamically rotates according to the 3D curvature of the substrate, keeping the printhead perpendicular to the local surface at all times. The platform is equipped with a piezoelectric printhead array (GH2220, Ricoh, Tokyo, Japan), consisting of two rows of 192 nozzles each. The default resolution is 300 dots per inch (DPI), corresponding to a droplet spacing of 84.5 µm. To enhance the density and uniformity of the sacrificial pattern, a precision printing strategy integrating image preprocessing and fine-axis displacement was developed (Figure 2c). Specifically, the original 1200 DPI pattern is decomposed via interlaced sampling into multiple 300 DPI sub-patterns. After printing each sub-pattern, the printhead is shifted by 21.1 µm along the print direction—matching the droplet pitch at 1200 DPI—until all sub-patterns are sequentially printed. This achieves a final effective resolution of 1200 DPI with a droplet spacing of 21.1 µm. To ensure the compactness and structural stability of the water-soluble sacrificial material, each printed layer is immediately cured using a 395 nm, 20 W UV light source. This print–cure cycle is repeated four times to build a uniform, densely packed sacrificial layer.

Next, a uniform metal layer is deposited over the entire substrate by ion sputtering for 800 s at a current of 20 mA. Finally, the substrate is immersed in water for 20 min to dissolve and to remove the water-soluble sacrificial material, resulting in the desired metal pattern.

### 2.2. Water-Soluble Sacrificial Ink Preparation

The water-soluble sacrificial ink used in this study consists of a photopolymerizable component, a water-soluble polymer, solvents, and a defoaming agent. For the photopolymerizable component, acryloylmorpholine (ACOM, Yinchang New Materials, Shanghai, China) was selected as the reactive monomer due to its high reactivity, low polymerization shrinkage, and excellent thermal stability. Under UV irradiation, ACOM rapidly forms a dense crosslinked network. Hydroxymethyl phenylphosphinic acid (HMPP, Curease Chemical, Shanghai, China) was used as the photoinitiator.

Polyvinylpyrrolidone (PVP, Zhonglian Chemical, Tianjin, China) was employed as the water-soluble polymer to control the solubility of the cured masking layer. As a typical water-soluble macromolecule, PVP exhibits excellent solubility and film-forming ability. The molecular weight and dosage of PVP directly affect the dissolution rate and complete lift-off behavior in water and also influence the structural integrity and mechanical properties of the cured film.

Dimethyl nylon acid (DBE, Zhonglian Chemical, Tianjin, China), a water-miscible and environmentally friendly organic solvent with low viscosity at room temperature (25 °C), was used as the primary solvent to ensure stable droplet formation during printing. Ethanol was introduced as a co-solvent to further reduce surface tension and improve droplet wettability and drying efficiency. The mass ratio of DBE to ethanol was 4:1. Additionally, industrial defoamer (LDS-800, Lujie Chemical Technology, Guangdong, China) was added to enhance ink stability.

**Figure 3:**
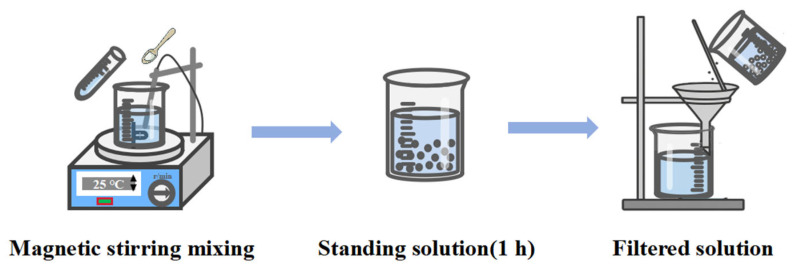
Ink preparation process.

The ink preparation process is illustrated in Figure 3. First, ACOM, PVP, and HMPP are sequentially added to a mixing vessel at room temperature according to the mass ratios listed in Table 1. After thorough dissolution via magnetic stirring, DBE, ethanol, and a defoaming agent are added and stirred until fully dissolved. The resulting mixture is then left to stand for 1 h to allow degassing, enhancing formulation stability. Finally, the solution is filtered to obtain the water-soluble sacrificial ink.

### 2.3. Droplet Observation Platform Setup

**Figure 4:**
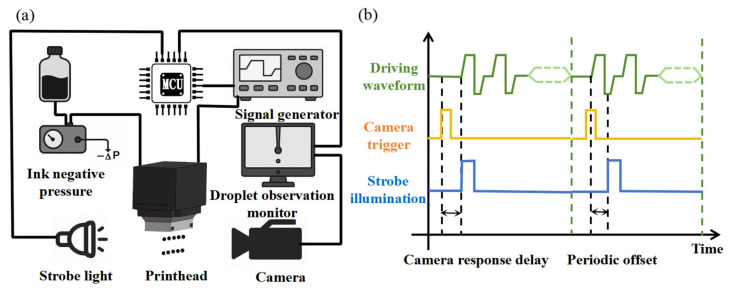
(**a**) Schematic of the droplet observation platform; (**b**) timing diagram of the printhead waveform synchronized with the imaging system.

To evaluate the actual jetting performance of the water-soluble sacrificial ink, a droplet observation platform was constructed, as illustrated in Figure 4a. The system consists of three main components: a printhead driving module, an imaging system, and an ink supply unit.

The printhead drive waveform is controlled by a signal generator (FY6300, FeelTech, Zhengzhou, China). The imaging system comprises a high-resolution camera (960 × 1280 pixels, MV-EM120M, Microvision Technology Co., Ltd., Xi’an, China) and a high-power LED strobe (XHP50.2, CREE, Durham, NC, USA) for capturing the evolution of droplet formation and monitoring the jetting behavior. The ink supply unit includes a precision negative pressure controller (FQFY-115C, FQ Electronics, Guangzhou, China) and a 5 mL dispensing syringe. The entire system is coordinated by an embedded control platform (STM32H743, STMicroelectronics, Geneva, Switzerland).

Given that the printhead operates at a jetting frequency of 1 kHz, with each jetting cycle lasting approximately 60 µs, while the camera’s maximum frame rate is 30 fps, a periodic delay strategy was implemented to capture the entire droplet formation process over multiple jetting cycles. Figure 4b illustrates the timing diagram of the printhead waveform synchronized with the imaging system. Upon receiving a trigger signal, the camera experiences a brief response delay before activating the strobe light. In the initial frame, the strobe is aligned with the printhead actuation to capture the onset of jetting. In subsequent frames, a fixed delay is introduced between triggers, based on the known ratio between jetting and imaging frequencies. This ensures that each frame captures a distinct phase of droplet motion, enabling complete reconstruction of the jetting process.

### 2.4. Testing and Characterization

A rotational rheometer (MCR 302, Anton Paar, Graz, Austria) and a contact angle meter (JC2000 D1, POWEREACH, Shanghai, China) were used to evaluate the rheological properties and surface tension of inks with varying PVP content. A 3D profilometer (KC-X1000, KathMatic, Nanjing, China) was employed to characterize the shape fidelity of the self-detaching water-soluble sacrificial material. A tensile testing machine (Aisry Instrument, Dongguan, China) was used to measure the adhesion strength between the metal layer and the substrate.

## 3. Results and Discussion

### 3.1. Inkjet Printability

During inkjet printing, ink is ejected from the nozzle under inertial forces. The resulting liquid column contracts due to surface tension and eventually breaks up into discrete droplets under the influence of viscosity. Throughout this process, the droplet undergoes complex dynamic changes governed by surface tension, fluid inertia, and viscosity. Therefore, the physical properties of the ink—particularly viscosity and surface tension—are critical for ensuring droplet formation stability and printability. Droplet behavior is typically characterized by dimensionless parameters such as the Reynolds number (*Re*), Weber number (*We*), and Ohnesorge number (*Oh*), which are defined as follows:

(1) $$Re=\frac{\nu \rho \alpha }{\eta }$$

(2) $$We=\frac{\nu^{2}\rho \alpha }{\gamma }$$

(3) $$Oh=\frac{\sqrt{We}}{Re}$$

where *υ* is the droplet velocity, *ρ* is the fluid density, *α* is the characteristic length (typically the nozzle diameter), *η* is the dynamic viscosity, and *γ* is the surface tension.

Among them, the Z number is widely used to assess inkjet printability, with values between 1 and 10 generally considered optimal for stable droplet formation without satellite droplets [32,33,34].

(4) $$Z=\frac{1}{Oh}$$

In this study, PVP was used as a water-soluble polymer, and its concentration had a significant influence on the solubility of the sacrificial material layer. Therefore, the effects of PVP mass fraction on the physical properties of the ink were systematically investigated, and the corresponding changes in the Z number were determined. These results provide theoretical support for optimizing inkjet printing process.

**Figure 5:**
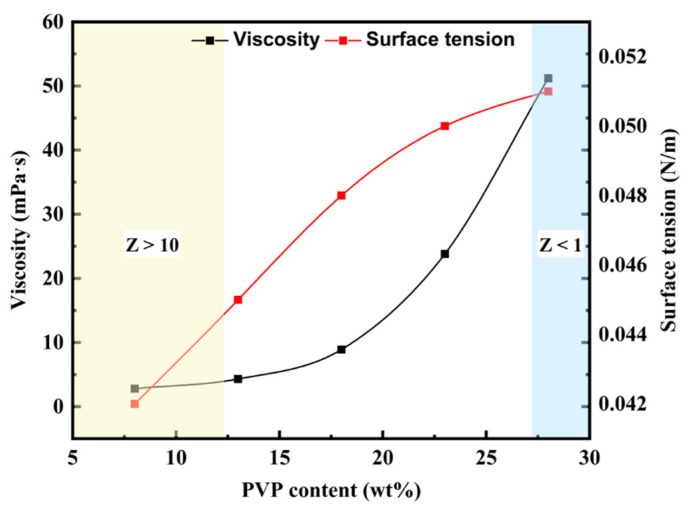
Results of viscosity and surface tension measurements on inks with different PVP content.

As shown in Figure 5, increasing the PVP content from 8 wt% to 26 wt% leads to a significant rise in ink viscosity—from approximately 5 mPa·s to nearly 50 mPa·s—exhibiting an exponential growth trend. This behavior is attributed to the entanglement of high-molecular-weight PVP chains in the solution, which increases internal flow resistance. In contrast, the surface tension increases only slightly (from 0.043 N/m to 0.051 N/m), primarily due to PVP’s mild inhibitory effect on the migration of other surfactant components. In the figure, the non-printable regions corresponding to Z > 10 (low-viscosity ink) and Z < 1 (high-viscosity ink) are marked with light yellow and light blue shading, respectively. These results indicate that only when the PVP mass fraction is within the range of approximately 13–26 wt% does the Z number fall within the printable window (1–10).

**Figure 6:**
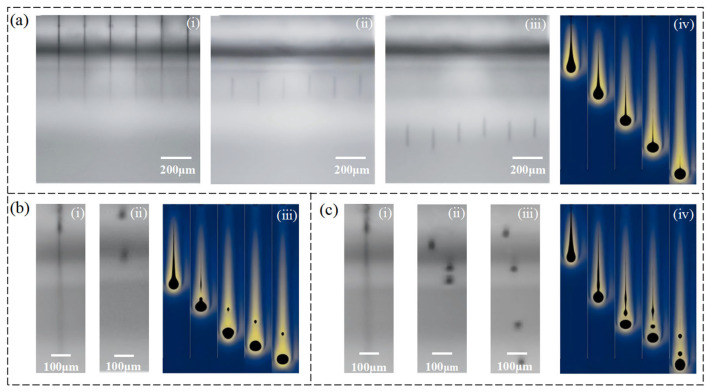
Comparison of experimental droplet jetting images and COMSOL simulation results for inks with different PVP concentrations: (**a**) 18 wt% (i–iii Droplet ejection process recorded by a high-speed droplet observation system; iv Simulated droplet dynamics modeled using COMSOL Multiphysics.), (**b**) 13 wt% (i–ii Droplet ejection process recorded by a high-speed droplet observation system; iii Simulated droplet dynamics modeled using COMSOL Multiphysics.), and (**c**) 23 wt% (i–iii Droplet ejection process recorded by a high-speed droplet observation system; iv Simulated droplet dynamics modeled using COMSOL Multiphysics.).

To comprehensively evaluate the effect of different PVP concentrations on inkjet performance, we conducted both experimental observations using a droplet visualization platform and numerical simulations via COMSOL. In the experimental section, three PVP concentrations within the printable window (13 wt%, 18 wt%, and 23 wt%) were selected for droplet imaging. As shown in Figure 6a(i)–(iii), the ink with 18 wt% PVP exhibited the most stable jetting behavior, with clear separation between the main droplet and tail, indicating optimal jetting conditions. In contrast, as shown in Figure 6b(i)–(ii), the 13 wt% ink, due to its low viscosity, produced unstable jets with poor droplet quality. For the higher viscosity ink (23 wt%), shown in Figure 6c(i)–(iii), delayed breakup and pronounced trailing were observed, reflecting reduced jetting stability.

To further elucidate these differences from a theoretical perspective, a 2D axisymmetric nozzle model was developed in COMSOL Multiphysics 6.1 using the two-phase-flow level-set method to simulate the transient droplet ejection process. Fluid parameters were set according to experimental measurements. The simulation covered a time span of 0–200 µs with a time step of 10 µs. The minimum mesh size in the interfacial region was set to 2.5 µm, and the level-set reinitialization parameter γ was set to 10. The simulation results were in good agreement with experimental observations. At an 18 wt% concentration (Figure 6a(iv)), the droplet broke off rapidly and stably, with smooth streamlines and a well-defined main droplet. At a 13 wt% (Figure 6b(iii)), premature necking and detachment of the main droplet were observed. At a 23 wt% (Figure 6c(iv)), the liquid filament was excessively stretched and the rear portion failed to break off effectively, indicating that surface viscous forces dominated the jetting process.

Combined analysis of both experimental and simulation results suggests that the ink formulation with an 18 wt% PVP achieves an optimal balance between physical properties and jetting dynamics and is therefore recommended as the optimal composition for inkjet printing in this study.

### 3.2. Forming Accuracy Characterization

**Figure 7:**
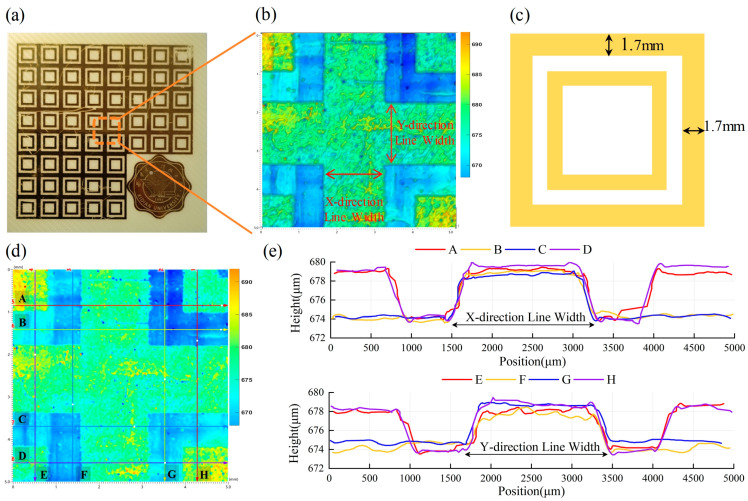
Analysis of forming accuracy. (**a**) Planar test sample; (**b**) surface profile measurement of the selected region; (**c**) standardized conductive pattern; (**d**) location of randomly selected profile lines; (**e**) cross-sectional profiles in the X and Y directions.

To assess the forming accuracy during the dissolution stage of the additive–subtractive manufacturing process, standardized conductive patterns (Figure 7c where closed-frame structures with a line width of 1.7 mm are shown) were fabricated on a planar substrate made of flame-retardant epoxy resin reinforced with fiberglass (FR4). A 3D surface profilometry scan was performed on the selected region (Figure 7a) to capture the post-dissolution morphology of the metal pattern. Figure 7b reveals a distinct height difference between the metal layer and the dissolved mask region. The color gradient reflects micron-level height variations, demonstrating the method’s high spatial resolution at the pattern edges.

To further assess the dimensional uniformity, eight representative cross-sectional profiles were randomly selected, as shown in Figure 7d, with four in the X direction (horizontal) labeled A–D and four in the Y direction (vertical) labeled E–H. These profile curves exhibit significant height transitions corresponding to the edges of the metal patterns (Figure 7e). The measured line widths of all metal traces range from 1681 μm to 1709 μm, with a maximum deviation within ±2%, showing excellent agreement with the designed width of 1700 μm. This corresponds to a forming accuracy better than ±20 μm. Moreover, no notable systematic differences were observed between the horizontal and vertical directions, indicating good isotropy during the dissolution process.

In summary, the experimental results demonstrate that the proposed additive–subtractive manufacturing strategy offers excellent pattern retention and dimensional precision, making it suitable for the controlled fabrication of high-accuracy metal patterns. 

### 3.3. Adhesion Characterization

To evaluate the adhesion strength of the conductive films fabricated in this work, pull-off tests were performed on flat ceramic substrates fully coated with the corresponding metal layers. This ensured uniform stress distribution and eliminated edge effects during the testing process. Two types of samples were prepared: (1) gold films deposited via ion beam sputtering and (2) silver films formed by inkjet printing of nanoparticle ink followed by sintering.

**Figure 8:**
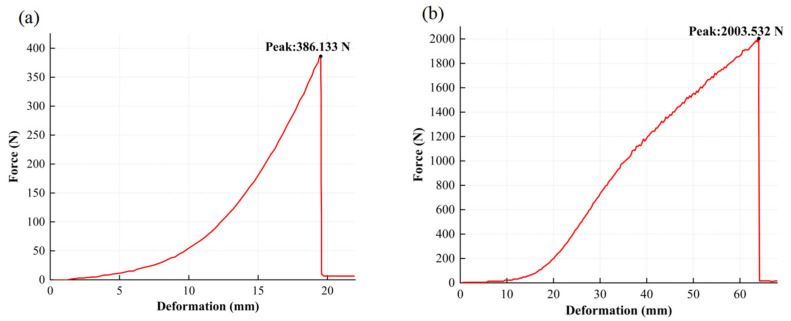
Adhesion test curve. (**a**) Silver films formed by inkjet printing of nanoparticle ink; (**b**) gold films deposited via ion beam sputtering.

A standard adhesion dolly with a radius of 10 mm was bonded to the metal surface using epoxy resin and then cured. The pull-off force was measured using an adhesion tester. The adhesion strength P was defined as $P=\frac{F}{S}$, where F is the peak force recorded at the moment of separation between the metal layer and the substrate during the pull-off test and S is the contact area of the adhesion dolly. As shown in the load–displacement curves in Figure 8a,b, the adhesion strength of the sputtered gold film reached 6.37 MPa, which is 5.2 times higher than that of the inkjet-printed silver film (1.23 MPa), indicating significantly stronger interfacial bonding in the former.

These results confirm that the proposed hybrid additive–subtractive manufacturing approach enhances the adhesion between the metal conductive layer and the substrate, without compromising the patterning resolution.

### 3.4. Fabrication of Conductive Patterns on Curved Rotational Surfaces and PET Film

**Figure 9:**
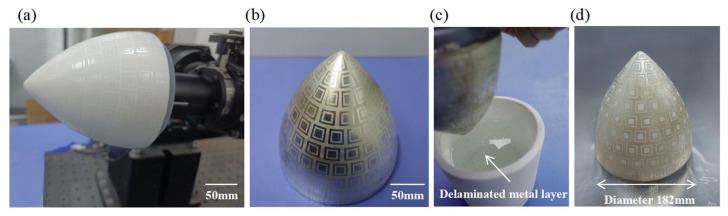
Curved conductive pattern fabrication via water-soluble sacrificial layer process. (**a**) Water-soluble sacrificial layer printing by inkjet (1200 DPI × 4 layers); (**b**) ion sputtering deposition of metal layer; (**c**) self-stripping of sacrificial layer in water (20 min immersion); (**d**) final conductive pattern on curved substrate.

To demonstrate the practicality of the proposed additive–subtractive manufacturing method based on a water-soluble sacrificial layer, conductive patterning was performed on a curved insulating ceramic substrate with a rotational surface radius of 91 mm. As shown in Figure 9, the complete process—including sacrificial material printing, metal deposition, and solvent-assisted removal—was successfully demonstrated as follows:

(a) First, the water-soluble sacrificial material was inkjet-printed in four successive layers at a high resolution of 1200 DPI to uniformly cover the curved surface of the insulating substrate. This high-precision printing ensured uniform material distribution, laying a solid foundation for the subsequent steps.
(b) Next, a metal layer was deposited over the entire printed surface using ion beam sputtering. The bombardment of high-energy ions enabled the metal atoms to embed into the substrate with high kinetic energy, forming a metal layer with strong interfacial adhesion.
(c) The sputtered sample was then immersed in water and left undisturbed for 20 min. During this period, the water-soluble sacrificial layer underwent a self-peeling process, selectively removing the metal film covering it.
(d) As a result, well-defined, firmly adhered, and intact metal patterns were successfully formed on the curved substrate.

These results strongly validate the effectiveness and feasibility of the proposed self-peeling, sacrificial-layer-based additive–subtractive manufacturing process for curved substrates, highlighting its potential for applications in complex surface fabrication.

**Figure 10:**
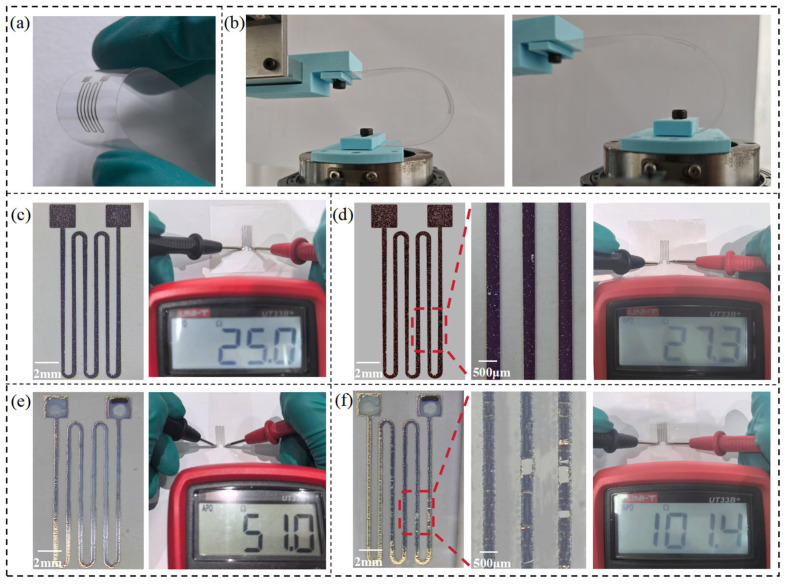
Flexible strain sensors fabricated on PET substrate. (**a**) Strain sensor fabricated on a PET film; (**b**) test setup of cyclic bending; (**c**) additive–subtractive sample before bending; (**d**) additive–subtractive sample after 500 bending cycles; (**e**) inkjet-printed sample before bending; (**f**) inkjet-printed sample after 500 bending cycles.

To further evaluate the electrical performance and adhesion durability of the conductive patterns fabricated by our proposed method, strain sensor samples were prepared on PET films using both conventional inkjet printing and the additive–subtractive method (Figure 10a). These samples were then subjected to 500 bending cycles, as illustrated in Figure 10b. Electrical resistance was measured before and after bending, and surface morphology was examined to assess changes in adhesion and electrical stability.

The fabrication procedures for both sample types are described as follows:

- **Additive–subtractive method samples:** PET films were first cleaned with ethanol to remove surface contaminants. A water-soluble sacrificial ink was then inkjet-printed at 1200 DPI in four successive layers, each cured under UV light for 20 s. A silver layer was subsequently deposited using a 100 W ion sputtering system for 900 s. Finally, the PET film was immersed in water for 20 min to dissolve the sacrificial layer and lift off the unwanted metal.
- **Inkjet-printed control samples:** The PET surface was treated with 100 W oxygen plasma for 15 min to improve hydrophilicity, thereby enhancing ink droplet adhesion and deposition precision. A silver nanoparticle ink was then inkjet-printed at 1200 DPI in four layers, followed by sintering under an 800 W infrared lamp for 45 s.

The results reveal a clear contrast between the two approaches. Before bending, the additive–subtractive sample exhibited a resistance of 25.0 Ω (Figure 10c), while the inkjet-printed sample showed a significantly higher resistance of 51.0 Ω (Figure 10d). This disparity is mainly attributed to the high sintering temperature (~180 °C) required for silver nanoparticle inks. Since PET substrates can withstand only up to 120 °C, the sintering process was incomplete, leading to poor conductivity in the inkjet-printed patterns. In contrast, the additive–subtractive method imposes no thermal constraints on the substrate, enabling the formation of well-adhered, highly conductive metal patterns even on temperature-sensitive materials like PET.

After 500 bending cycles, the inkjet-printed samples exhibited severe surface damage and cracking (Figure 10f), accompanied by a dramatic increase in resistance to 101.4 Ω, indicating significant performance degradation. In contrast, the additive–subtractive samples maintained intact morphology with no visible defects (Figure 10e), and their resistance increased only slightly to 27.3 Ω. These results clearly demonstrate that our method offers superior adhesion and electrical stability.

## 4. Conclusions

In this study, an additive–subtractive manufacturing method based on a water-soluble sacrificial layer is proposed to address inkjet printing and the poor adhesion of inkjet-printed conductive patterns on curved substrates. Droplet observation experiments combined with numerical simulations demonstrated that, by tuning the solid content of the main component in the sacrificial ink, the viscosity and surface tension could be effectively optimized, enabling stable and high-quality jetting behavior. Experimental results showed that, after the dissolution of the sacrificial layer, the process achieved a patterning resolution better than ±20 μm. Pull-off tests further confirmed the superior adhesion performance of the proposed method, with the adhesion strength of the fabricated metal layer being 5.2 times higher than that of conventional inkjet-printed nanoparticle silver films. Moreover, this process was successfully applied to the fabrication of conductive patterns on a three-dimensional curved surface with a curvature radius of 91 mm, demonstrating its feasibility and broad application potential in complex-surface electronics manufacturing.

Looking ahead, the method described herein will undergo further optimization. We aim to extend the technology to industrial-level applications, including conformal 3D printing on complex geometries.

## Figures and Tables

**Table 1:** Ink composition and mass ratios.

Element	DBE	ACOM	PVP	Ethanol	HMPP	Defoamer
wt%	36–44%	30%	13–23%	9–11%	1.5%	1%

## Data Availability

The raw data supporting the conclusions of this article will be made available by the authors on request.

## Abbreviations

The following abbreviations are used in this manuscript:

DPI	Dots per inch
AM	Additive manufacturing
UV	Ultraviolet
PEEK	Polyetheretherketone
ITO	Indium tin oxide
ACOM	Acryloylmorpholine
HMPP	Hydroxymethyl phenylphosphinic
PVP	Polyvinylpyrrolidone
DBE	Dimethyl nylon
PET	Polyethylene glycol terephthalate
